# A multidimensional data-driven approach to surgical plan optimization and postoperative residual tumor prediction in ovarian cancer

**DOI:** 10.3389/fimmu.2025.1705428

**Published:** 2025-12-17

**Authors:** Lin Yang, Tianhui He, Ping Yu, Chunliang Shang, Jing Wang, Qinkun Sun, Yi Xie, Jianling Yang, Hongyan Guo

**Affiliations:** 1 Department of Obstetrics and Gynecology, Peking University Third Hospital, Beijing, China; 2 Mass General Cancer Center, Massachusetts General Hospital, Harvard Medical School, Boston, MA, United States; 3 Center of Basic Medical Research, Institute of Medical Innovation and Research, Peking University Third Hospital, Beijing, China

**Keywords:** high-grade serous ovarian cancer, residual tumor prediction, ascites immune immune-based cytoreduction prediction for HGSOC profiling, deep learning model, immune exhaustion biomarkers

## Abstract

**Backgrounds:**

Ovarian cancer represents a deadly gynecological malignancy, with surgical treatment being a key component of its management. We sought to integrate clinical characteristics and ascites immune microenvironment features into a deep learning model to predict postoperative residual tumor status and assist in surgical decision-making.

**Methods:**

118 FIGO III/IV high-grade serous ovarian cancer (HGSOC) patients treated at Peking University Third Hospital (2019-2024) were enrolled. Clinical characteristics, surgical methods, and postoperative residual tumor status were collected. Ascites samples were processed via density gradient centrifugation and flow cytometry. Deep learning model was built by fusing clinical and immune data, and its performance was validated under a gradient of feature quantities (5–45 features) to optimize feature selection. Model performance was comprehensively evaluated on a test set (20% of the dataset) using metrics including accuracy, precision, recall, and F1 score, and compared with traditional machine learning models (random forest, XGBoost, et al). Confusion matrices and probability heatmaps were used for visual analysis. For model interpretability, we presented feature importance and results from SHAP analysis.

**Results:**

Our model achieved 70.83% accuracy, 71.21% precision, 70.83% recall, and 70.89% F1 score on the test set, outperforming traditional machine learning models: random forest (accuracy: 64.6%, precision: 65.1%, recall: 64.6%, F1 score: 66.4%), XGBoost (accuracy: 66.7%, precision: 67.0%, recall: 66.7%, F1 score: 66.6%), and logistic regression (accuracy: 58.3%, precision: 59.0%, recall: 58.4%, F1 score: 58.2%). It demonstrated strong performance in identifying high-risk R2 cases but showed limitations in differentiating between R0 and R1 statuses. Probability heatmaps visualized the distribution of R0, R1, and R2 probabilities under different surgical methods, facilitating intuitive clinical reference. Interpretability analysis via permutation feature importance and SHAP highlighted the critical role of surgical methods and specific immune microenvironment features in predictive outcomes.

**Conclusion:**

This study developed a novel deep learning-based model to predict postoperative residual tumor probability, integrating clinical and immune microenvironment data. While the model excelled in identifying high-risk cases (e.g., R2), further optimization is needed to improve R0 and R1 differentiation. Future research should expand datasets and integrate multi-omics data to enhance predictive accuracy and clinical applicability.

## Introduction

Ovarian cancer is one of the deadliest diseases among gynecological malignancies (1–3), with its treatment primarily involving surgery and chemotherapy. Surgical treatment, particularly cytoreductive surgery, is the cornerstone of ovarian cancer management (4, 5). The primary goal is to achieve an R0 state (no visible residual tumor) by thoroughly excising tumor tissues. However, the choice of surgical approaches currently relies heavily on the clinical experience of physicians (6), lacking unified standards and scientific evidence (7). Achieving an R0 state during surgery is a critical factor affecting patient prognosis. Yet, in practice, real-time determination of R0 status is often challenging, leading to uncertainties in surgical outcomes. Additionally, The surgical plan primarily relies on laparoscopic exploration, with the next steps determined based on the extent of tumor dissemination observed during the procedure. However, this approach overlooks the role of multidimensional data such as the immune microenvironment and molecular markers, thereby limiting the personalization and accuracy of surgical strategies (5).

In recent years, with the rapid advancement of precision medicine, increasing evidence suggests that the immune microenvironment (e.g., the composition of immune cells in ascites) plays a pivotal role in the occurrence, progression, and therapeutic response of ovarian cancer (8). For example, the immune cell composition in ascites may reflect tumor immune evasion mechanisms. However, the application of such multidimensional data in clinical decision-making remains limited, particularly in surgical planning, where no systematic integration methods have been developed. Effectively applying these data to clinical practice has become a key research direction (9).

Simultaneously, the rapid development of artificial intelligence (AI) technology has brought new opportunities to the medical field (10–12). AI can integrate multidimensional data (e.g., clinical characteristics, immune microenvironment) to provide scientific support for clinical decision-making. For instance, deep learning models can analyze complex nonlinear relationships to predict patient responses to treatment and prognosis, thereby assisting clinicians in formulating personalized treatment plans (13). However, no studies currently utilize AI technologies to predict and recommend surgical approaches for ovarian cancer, particularly in predicting the probability of postoperative residual tumor (R0/NR0). Existing research predominantly focuses on analyzing single types of data, lacking integration of multidimensional data to comprehensively reflect patient conditions and surgical risks (14–16).

Against this background, this study aims to develop a deep learning model that integrates patients’ clinical characteristics (e.g., age, stage, initial serum CA125 levels) and immune microenvironment data (e.g., immune cell composition in ascites). This model will be used to predict the probability of postoperative residual tumor under different surgical approaches. By providing a quantitative assessment of residual tumor risk for various surgical options, the study seeks to offer clinicians objective reference information during surgical decision-making. This approach introduces a data-driven method for formulating ovarian cancer surgical plans, which can help reduce the variability in surgical decisions and potentially improve surgical outcomes.

## Methods

### Study subjects

**Figure 1:**
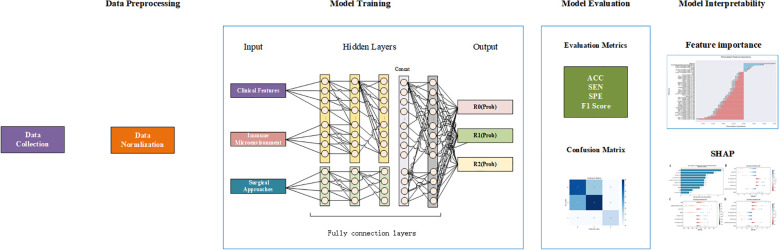
Workflow of the integrated deep learning framework. First,data acquisition from clinical records and ascites samples; Second, data processing; Third, model development and training;Fourth, prediction and evaluation of residual tumor probability (R0/R1/R2). Finally, the model interpretability.

This study focuses on patients with newly diagnosed high-grade serous ovarian cancer (HGSOC) at FIGO stage III/IV, who completed first-line treatment at Peking University Third Hospital between May 2019 and March 2024, and all patients had ascites. To ensure homogeneity of the study cohort, patients with other types of ovarian benign or malignant diseases, systemic infections, hematologic disorders, liver and kidney diseases, or other malignancies were excluded. Additionally, patients who had received any anti-tumor treatments such as surgery, chemotherapy, radiotherapy, or immunotherapy within the past five years, as well as those lost to follow-up during the study, were also excluded. Ultimately, 118 patients met the inclusion criteria and participated in this study, The workflow of this research shown in **Figure 1**.

The study was conducted in accordance with the Declaration of Helsinki (as revised in 2013) and approved by the designated institution (the Ethics Committee of Peking University Third Hospital) with the approval number IRB00006761-M2019291. Given the retrospective and anonymized characteristics of this study, the requirement for individual informed consent was waived.

### Data collection

This study collected the basic clinical characteristics of all HGSOC patients, including age, FIGO stage, preoperative serum CA125 levels, ascites volume, surgical approach, and postoperative residual tumor status. Postoperative residual tumor status was classified into three categories based on surgical outcomes: no visible residual tumor (R0), residual tumor with a maximum diameter ≤ 1 cm (R1), and residual tumor with a maximum diameter > 1 cm (R2). R1 and R2 are collectively referred to as non-R0 (NR0).

### Immune microenvironment features for model input

The following 46 immune cell subsets, quantified via flow cytometry from ascites samples, were included as features in the machine learning models: CCR7+CD45RA+ CD4+T cell, CCR7+CD45RA-CD4+T cell, CCR7-CD45RA-CD4+T cell, CCR7-CD45RA+CD4+T cell, CD28+CD4+T cell, CD57+CD4+T cell, PD-1+CD4+T cell, Tim-3+CD4+T cell, CD28+CD57-CD4+T cell, CD28+CD57+CD4+T cell, CD28-CD57-CD4+T cell, CD28-CD57+CD4+T cell, PD-1-CD28+CD4+T cell, PD-1+CD28+CD4+T cell, PD-1-CD28-CD4+T cell, PD-1+CD28-CD4+T cell, PD-1-CD57-CD4+T cell, PD-1-CD57+CD4+T cell, PD-1+CD57-CD4+T cell, PD-1+CD57+CD4+T cell, CCR7+CD45RA+CD8+T cell, CCR7+CD45RA- CD8+T cell, CCR7-CD45RA-CD8+T cell, CCR7-CD45RA+CD8+T cell, CD28+CD8+T cell, CD57+CD8+T cell, PD-1+CD8+T cell, Tim-3+CD8+T cell, CD28+CD57-CD8+T cell, CD28+CD57+CD8+T cell, CD28-CD57-CD8+T cell, CD28-CD57+CD8+T cell, PD-1-CD28+CD8+T cell, PD-1+CD28+CD8+T cell, PD-1-CD28-CD8+T cell, PD-1+CD28-CD8+T cell, PD-1-CD57-CD8+T cell, PD-1-CD57+CD8+T cell, PD-1+CD57-CD8+T cell, PD-1+CD57+CD8+T cell.

### Sample collection and processing

Ascites samples were collected during surgery and processed within one hour of collection. The samples were first centrifuged at 2,000 g for 10 minutes at 4 °C, followed by density gradient centrifugation using Ficoll (density 1.077, GE Healthcare) and PBS in a 1:1.5 ratio to separate mononuclear cells. The isolated cells were washed twice with PBS and then centrifuged at 500 g for 5 minutes. Cell counting was performed manually, and the cells were stored at 0°C to ensure the staining process was completed promptly.

Surgical procedures were categorically defined as follows:

Primary Debulking Surgery (PDS): Cytoreductive surgery performed as the initial intervention, with the intention of achieving complete resection.

Neoadjuvant Chemotherapy followed by Interval Debulking Surgery (NACT+IDS): Patients who received 3–4 cycles of platinum-based chemotherapy prior to undergoing surgery.

Laparoscopic Biopsy Only: A diagnostic procedure where only a biopsy was taken, without an attempt at cytoreduction, typically due to extensive, unresectable disease or significant patient co-morbidities. This categorization was confirmed by review of operative notes and surgical reports.

### Flow cytometry detection

Under light protection at room temperature, 1×10^6 cells were stained for 15 minutes using specific monoclonal antibodies (mAbs). After staining, cells were fixed with 1% paraformaldehyde. Flow cytometry analysis was performed using a CytoFLEX S (Beckman Coulter) device, and data were analyzed using Cytoexpert v. 2.3 software.

### Feature selection and rationale

Feature selection was conducted using a hybrid approach combining clinical knowledge and data-driven analysis. Firstly, all clinically established prognostic factors (e.g., Age, FIGO Stage, serum CA125) were retained due to their face validity. Secondly, for the high-dimensional flow cytometry data, we employed a two-step filtering process: (1) Univariate analysis (Kruskal-Wallis test) was used to identify immune cell subsets with differential abundance across R0/R1/R2 groups (p < 0.2 was used as a liberal threshold to avoid pre-maturely excluding potential signals). (2) Subsequently, these pre-selected features, along with the core clinical variables, were input into the model, which inherently performs regularized feature selection through its architecture and dropout layers. This process ensures that the final model relies on a robust set of features that are both clinically relevant and statistically informative.

### Data preprocessing, model construction, and training

The data used in this study were derived from clinical records of ovarian cancer patients, including clinical features, immune microenvironment data, and postoperative residual tumor status. All preprocessing was performed in a leak-proof manner within the training partitions of cross-validation to prevent information leakage. First, the feature data were standardized to have a mean of 0 and a standard deviation of 1, reducing the impact of dimensional differences on model training and ensuring the quality of the input data. The standardized data were then controlled within a reasonable range, further improving the model’s stability.

The deep learning model was explicitly architected to integrate these diverse data types effectively. Its core structure consists of parallel processing branches that converge into a unified prediction. All preprocessed clinical and selected immune features are processed through a dedicated feature extraction branch, which employs a sequence of fully connected layers, each followed by Batch Normalization and a LeakyReLU activation function to capture complex non-linear relationships. Simultaneously, the categorical Surgical Method feature is processed through a separate embedding layer, which transforms it into a dense, continuous vector representation. This embedding allows the model to learn nuanced, non-linear associations between the type of surgery and patient-specific factors, rather than treating it as a simple categorical variable. The outputs from the clinical/immune branch and the surgical method embedding branch are then concatenated, forming a rich, fused feature representation. This combined vector is subsequently passed through additional fully connected layers for high-level interaction modeling before a final softmax output layer generates probability distributions for the three residual tumor outcomes (R0, R1, R2).

During the training phase, the model was optimized to minimize a weighted categorical cross-entropy loss function. The class weights were set to be inversely proportional to their frequencies in the training data, a deliberate strategy to counteract the inherent class imbalance between the R0, R1, and R2 groups. The RMSprop optimizer was employed with an initial learning rate of 0.001, which was dynamically reduced upon plateau of the validation loss. Training proceeded for a maximum of 100 epochs with a batch size of 32, and an early stopping mechanism with a patience of 10 epochs was enforced to halt training and prevent overfitting when no further improvement in validation performance was observed.

### Surgical method embedding mechanism

**Figure 2:**
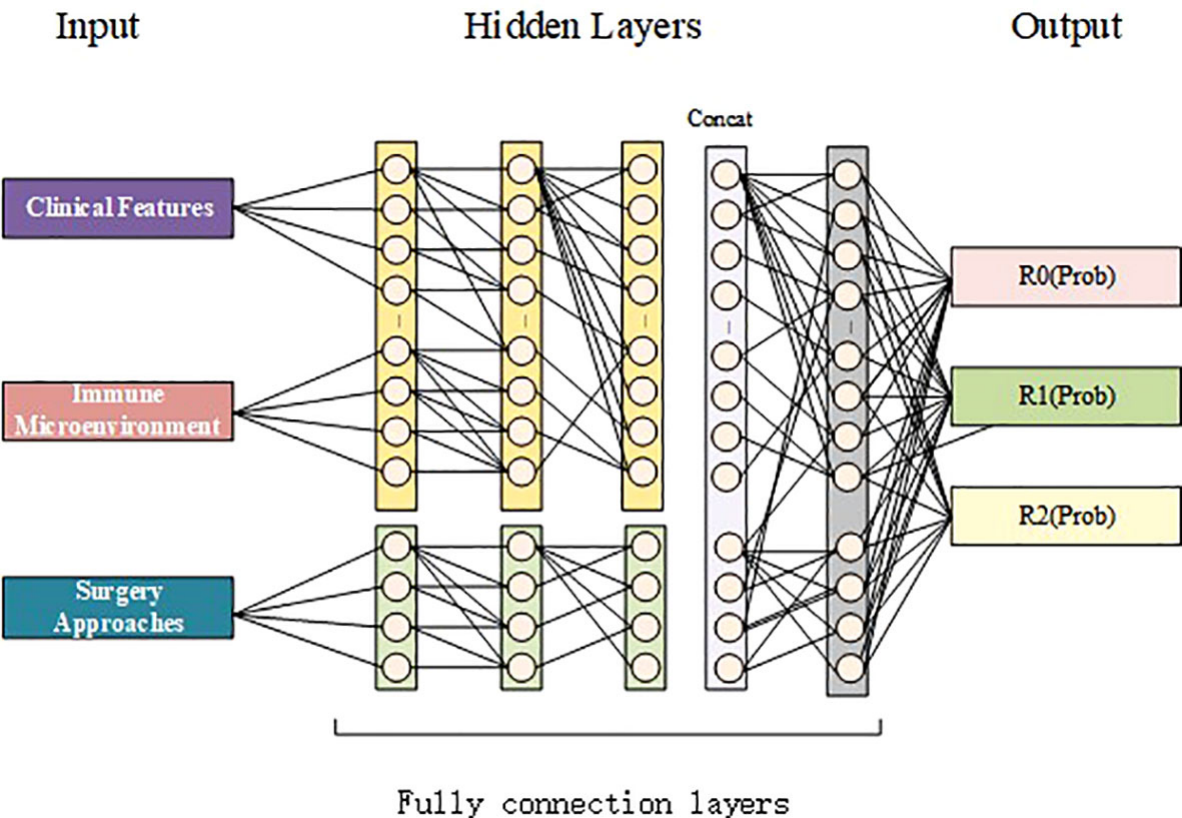
Architecture of the deep learning-based residual tumor predictor. The model integrates clinical features and Immune microenvironment features and surgery method to predict the probability of postoperative residual tumor status (R0, R1, or R2).

The surgical approach was processed through an embedding layer, which transforms the categorical surgical method into a continuous vector representation (as shown in **Figure 2**). The mechanistic rationale for this design is that it allows the model to learn nuanced, non-linear relationships between the chosen surgical procedure and the patient’s specific clinical and immune profile. For instance, the model can learn that the effectiveness of Primary Debulking Surgery (PDS) is not fixed but varies considerably depending on a patient’s tumor stage and immune status. The embedding layer captures these complex interactions by projecting the surgical method into a latent space where geometrically similar vectors correspond to surgical strategies with similar predicted outcomes for similar patient types. This is a more powerful representation than simple one-hot encoding, as it enables the model to generalize based on learned functional similarities between surgical contexts.

### Model training

During the model training phase, a weighted cross-entropy loss function was used to address the class imbalance issue, with the weights calculated based on the sample distribution of each class in the training set. The RMSprop optimizer was chosen, combined with a learning rate scheduler to dynamically adjust the learning rate. The model was trained for 100 epochs, with the training data processed in batches (batch size = 32) for each epoch, and the loss value was calculated. Model parameters were updated via backpropagation and gradient clipping, while early stopping was applied to prevent overfitting. Training was terminated early if the validation loss did not decrease for 10 consecutive epochs. To address the class imbalance between R0, R1, and R2 groups, we employed multiple strategies. During model training, a weighted cross-entropy loss function was used, where the class weights were inversely proportional to their frequencies in the training set, thereby penalizing misclassifications in the minority R1 and R2 groups more heavily. Furthermore, the model’s performance was evaluated using weighted averages for precision, recall, and F1-score, which provides a more realistic assessment of its utility across all classes compared to accuracy alone.

### Model evaluation and validation

To ensure a robust and unbiased evaluation of the model’s performance given the modest sample size, we implemented a rigorously designed validation framework. The entire dataset was first stratified by the target variable (residual tumor status) and then partitioned into a temporary training set (80% of the data) and a held-out test set (the remaining 20%). This held-out test set was strictly reserved for the final performance assessment and was never used in any model development or feature selection steps.

To optimize model hyperparameters and obtain a stable performance estimate, we employed a nested cross-validation (CV) scheme exclusively on the temporary training set. The outer loop was a 5-fold stratified CV, which creates five different train/validation splits. Within each fold of the outer loop, an inner 5-fold stratified CV was performed on the training split to tune hyperparameters (e.g., learning rate, dropout rate, L1 regularization strength). The model was then retrained on the entire training split of the outer fold using the best hyperparameters and evaluated on the corresponding validation fold. This process yields five performance estimates (e.g., for accuracy, F1-score) that account for the variance introduced by data partitioning and hyperparameter tuning.

The final model, intended as a potential prototype for future validation, was trained on the entire temporary training set (100% of the 80% partition) using the hyperparameters that demonstrated the best average performance during the inner CV loops. The generalization performance reported in this study (**Table 1**) is the model’s performance on the completely unseen, held-out test set (20% of the data). This stringent approach provides a more reliable and pessimistic estimate of how the model would perform on new data from a similar patient population, thereby strengthening the credibility of our findings despite the sample size limitation.

**Table 1:** Performance metrics for different models.

Model	Accuracy (95%CI)	Precision (95%CI)	Recall(95%CI)	F1Score (95%CI)	ROC
Deep learning model	0.7083(0.5921-0.8243)	0.7121(0.5987-0.8263)	0.7083(0.5922-0.8243)	0.7089(0.5933-0.8253)	0.801(0.690-0.912)
LightGBM	0.695(0.578–0.812)	0.699(0.582–0.816)	0.695(0.578–0.812)	0.696(0.579–0.813)	0.789(0.675–0.903)
SVM	0.687(0.571–0.803)	0.689(0.573–0.805)	0.687(0.571–0.803)	0.688(0.572–0.804)	0.781(0.667–0.895)
Random forest	0.646(0.5253-0.7674)	0.651(0.5301-0.7721)	0.646(0.5254-0.7678)	0.664(0.5439-0.7851)	0.752(0.635-0.869)
XGBoost	0.667(0.5466-0.7887)	0.670(0.5498-0.7914)	0.667(0.5464-0.7886)	0.666(0.5454-0.7871)	0.771(0.656-0.886)
Logistic regression	0.583(0.4593-0.7075)	0.590(0.4665-0.7149)	0.584(0.4606-0.7084)	0.582(0.4584-0.7067)	0.698(0.575-0.821)

### Model explainability analysis

To gain a deeper understanding of the model’s reliance on input features and its decision-making process, this study employed the Permutation Feature Importance method for model explainability analysis. Permutation feature importance assesses the importance of a feature by randomly shuffling its values and observing changes in model performance. Specifically, if a feature is critical to the model’s prediction, shuffling its values will lead to a significant drop in performance. Conversely, if a feature has minimal impact on the model’s prediction, its performance will not be significantly affected.

For the test set, we performed 10 random permutations for each feature and computed the change in model performance (using accuracy by default), recording the importance score for each permutation. Through permutation feature importance analysis, we identified the key features that have the most significant impact on the model’s predictions. The analysis results not only help validate the model’s reasoning but also provide valuable insights for clinicians, helping them understand the basis of the model’s decisions and improving their ability to develop personalized surgical plans. To further decompose the model’s decision-making mechanism at the individual sample level and quantify the directional impact of features (a key complement to permutation importance, which lacks contribution polarity), we implemented SHAP analysis. Rooted in cooperative game theory, SHAP assigns a Shapley value to each feature for every sample, representing the feature’s marginal contribution to shifting the model’s prediction from a global baseline (average predicted probability of R0/R1/R2 across all samples) to the sample-specific outcome.

### Statistical analysis

Statistical analyses were performed to characterize the patient cohort and, where applicable, to provide context for the machine learning predictions. For comparisons of continuous variables across the three residual tumor status groups (R0, R1, R2), the Kruskal-Wallis H-test was employed, as the data distributions were assessed for normality using the Shapiro-Wilk test and found to be non-parametric. *Post-hoc* pairwise comparisons were conducted using Dunn’s test with a Bonferroni correction for multiple comparisons. For categorical variables, between-group differences were assessed using the Chi-squared test or Fisher’s exact test when expected cell counts were less than 5.

It is important to note that the primary objective of this study was the development and evaluation of a predictive model. Therefore, the aforementioned statistical tests were used for descriptive purposes and to explore potential associations within the cohort, rather than as the main analytical focus. Survival analysis was not an endpoint of this study, as the model’s purpose is to predict the immediate surgical outcome (residual disease status), not long-term survival. All statistical analyses were conducted using Python (version 3.9) with the Scipy stats library (version 1.11.0), and a two-sided p-value of less than 0.05 was considered statistically significant.

The performance metrics of the deep learning model (Accuracy, Precision, Recall, F1-Score) are reported with their 95% confidence intervals (95% CI), calculated using the bootstrapping method with 1,000 iterations on the held-out test set. This provides an estimate of the uncertainty around the point estimates of model performance.

For between-group comparisons of baseline characteristics, continuous variables are presented as mean ± standard deviation or median (interquartile range), and categorical variables as frequency (percentage). As previously stated, the Kruskal-Wallis H-test was used for continuous variables and the Chi-squared or Fisher’s exact test for categorical variables. Results of these inferential tests are reported with their corresponding test statistic and p-value.

## Results

### Patient characteristics

A total of 214 consecutive patient records with high-grade serous ovarian cancer (HGSOC) were initially screened for eligibility. After applying the exclusion criteria, 96 patients were excluded for the following reasons: non-HGSOC histology (n=42), absence of ascites (n=25), incomplete clinical or flow cytometry data (n=18), receipt of prior anti-tumor therapy within five years (n=7), and loss to follow-up (n=4). Consequently, 118 patients met all inclusion criteria and constituted the final study cohort for model development and analysis.

The key baseline clinical characteristics of these 118 patients are summarized in **Table 2**. The mean age was 58.75 ± 11.01 years, with an equal distribution of FIGO Stage III (50.00%) and Stage IV (50.00%) disease. The majority of patients (66.95%) had an ascites volume of ≥2000 ml. Regarding surgical management, 39.83% underwent Primary Debulking Surgery (PDS), 43.22% received Neoadjuvant Chemotherapy followed by Interval Debulking Surgery (NACT+IDS), and 16.95% underwent Laparoscopic biopsy only. Postoperative residual tumor status was R0 in 46.61%, R1 in 29.66%, and R2 in 23.73% of patients.

**Table 2:** Clinical features of included patients.

Characteristics	Ascites (n=118)
Age (years)	58.75 ± 11.01
FIGO stage	
phase III	59 (50.00%)
phase IV	59 (50.00%)
serum CA125 (U/mL)	1136 (517.65, 2196)
Ascites	
<2000ml	39 (33.05%)
≥2000ml	79 (66.95%)
Surgical procedure	
PDS	47 (39.83%)
NACT+IDS	51 (43.22%)
Laparoscopic biopsy	20 (16.95%)
Postoperative residual tumor size	
R0	55 (46.61%)
R1	35 (29.66%)
R2	28 (23.73%)

### Data feature description

The main clinical features and distribution of the patients are shown in **Table 2**.

### Model performance

The deep learning model’s performance was comprehensively evaluated on the test set, which is a random 20% subset of the data. As shown in **Table 1**, The deep learning model achieved a test accuracy of 0.708. The precision was 0.712, reflecting the proportion of true positive samples among all predicted positive samples. The recall was 0.708, indicating the model’s ability to correctly identify positive samples. The F1 score, which balances precision and recall, was 0.709, demonstrating the model’s robust classification performance. These metrics were calculated using weighted averages to address the class imbalance issue and ensure fair evaluation across all categories. The random forest model achieved an accuracy of 0.646 recision of 0.651, recall of 0.646, and F1 score of 0.664; the XGBoost model yielded an accuracy of 0.667, precision of 0.670, recall of 0.667, and F1 score of 0.666 while the logistic regression model performed the least effectively with an accuracy of 0.583, precision of 0.590, recall of 0.584, and F1 score of 0.582.

In addition to the deep learning model, we expanded our comparison to include two other prominent machine learning algorithms, LightGBM and Support Vector Machine (SVM with RBF kernel), as well as a baseline clinical model. This baseline model was constructed using only the core clinical predictors (FIGO Stage and serum CA125 level) through logistic regression, representing a simplified traditional diagnostic approach.

The results, now included in the updated **Table 1** and **Supplementary Figure S1**, demonstrate that our deep learning model achieved superior performance across all metrics, including the highest ROC-AUC of 0.801. While LightGBM and SVM showed competitive results, they were consistently outperformed by the deep learning model. Most notably, the deep learning model showed a substantial improvement over the clinical baseline model (ROC-AUC: 0.801 *vs*. 0.663), underscoring the significant added value of integrating the ascites immune microenvironment data for predicting postoperative residual tumor status.

### Confusion matrix and model performance for deep learning

**Figure 3:**
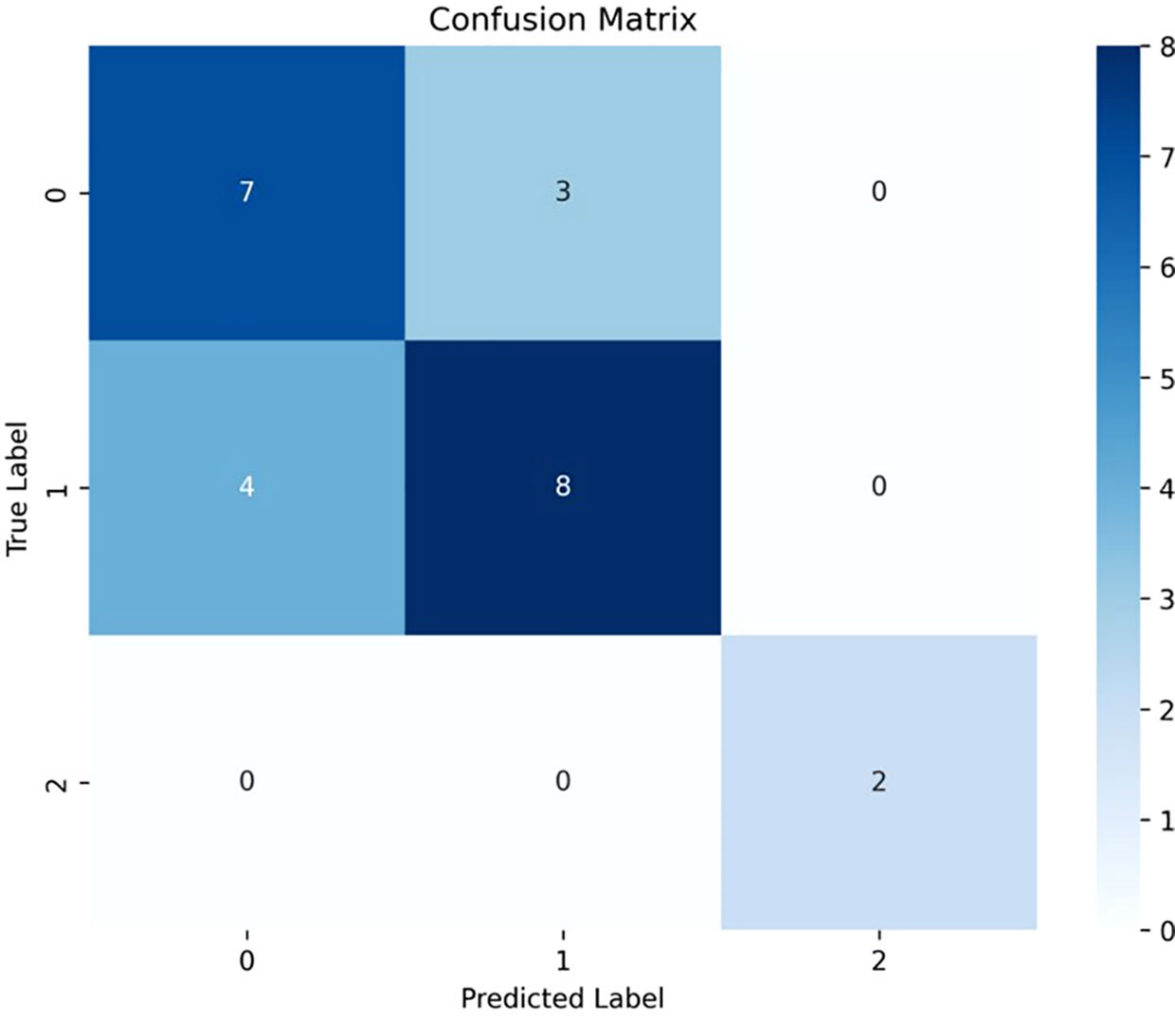
Confusion matrix of residual tumor prediction. Diagonal values indicate correct predictions for R0 (7/10), R1 (8/12), and R2 (2/2) classes.

As shown in **Figure 3**, the confusion matrix indicates that the Deep learning model correctly predicted 7 out of 10 R0 cases (no visible residual tumor), 8 out of 12 R1 cases (residual tumor ≤ 1 cm), and correctly predicted all 2 R2 cases (residual tumor > 1 cm). While the model performed excellently in distinguishing R2 cases, there is still room for improvement in distinguishing between R0 and R1 cases.

### Probability heatmap for clinical interpretation

**Figure 4:**
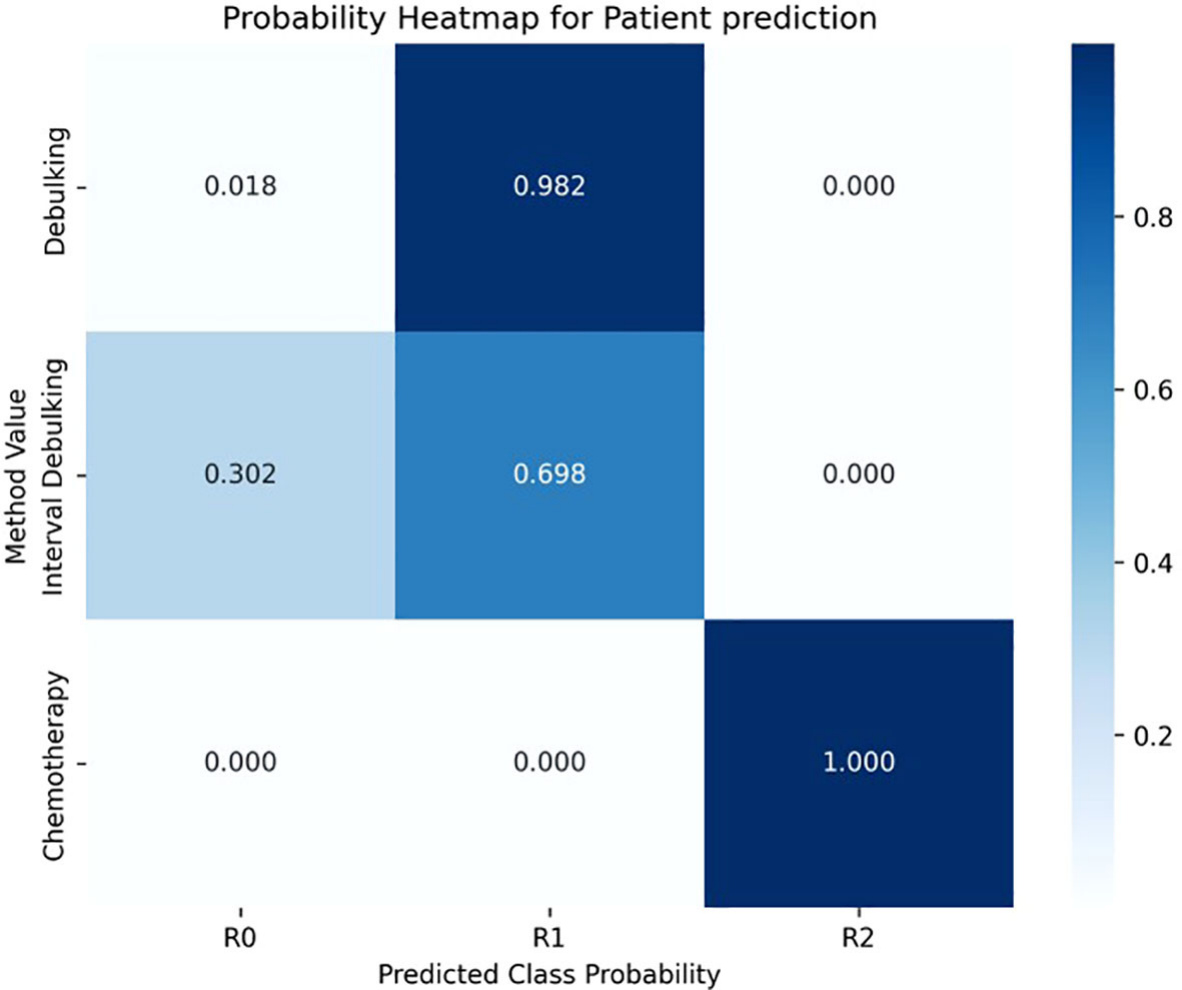
Probability heatmaps illustrating the predicted probabilities of achieving R0 (no visible residual tumor), R1 (residual tumor ≤1 cm), or R2 (residual tumor >1 cm) status under different surgical methods.

To facilitate clinical application and enhance the interpretability of the model’s predictions, probability heatmaps (**Figure 4**) were generated to visually present the predicted probabilities of achieving R0, R1, or R2 status under different surgical methods. These heatmaps illustrate how various surgical approaches affect the likelihood of complete tumor removal (R0) versus residual tumor presence (R1 or R2). By providing actionable insights, these visualizations enable clinicians to tailor surgical strategies based on individual patient circumstances, ultimately supporting the development of personalized treatment plans.

### Model interpretability analysis results

**Figure 5:**
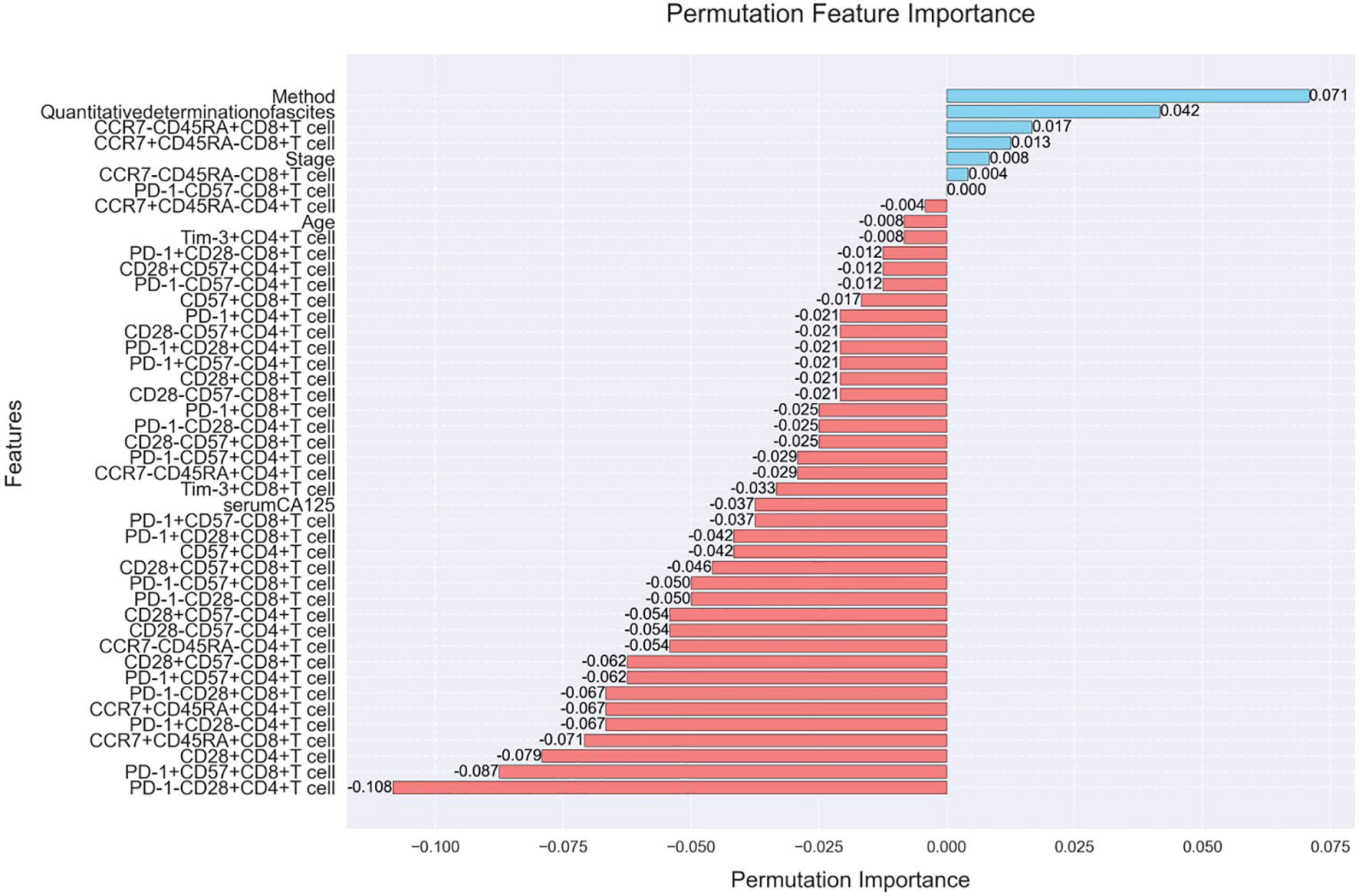
Permutation feature importance analysis of predictive features.

**Figure 6:**
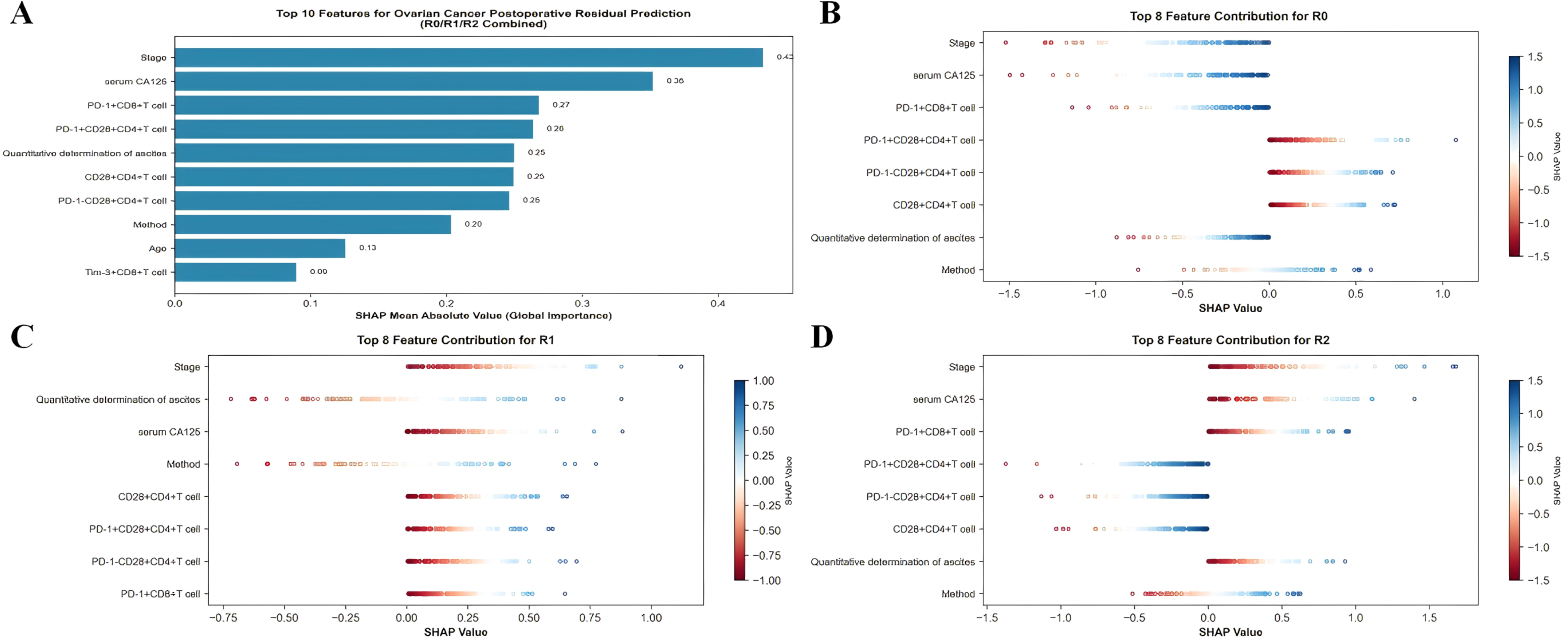
The SHAP results for R0/R1/R2. **(A)** Top 10 Features for Ovarian Cancer Postoperative Residual Prediction(R0/R1/R2 combined). **(B)** Top 8 Feature Contribution for R0. **(C)** Top 8 Feature Contribution for R1. **(D)** Top 8 Feature Contribution for R2.

The analysis results indicate that several features have a significant positive or negative impact on the model’s predictions (**Figure 5**). Among the positive influences, the surgical method (Method) had the highest importance score of 0.071, suggesting that it plays the most significant role in predicting the probability of postoperative residual tumor. Additionally, CCR7-CD45RA+CD8+ T cells and CCR7+CD45RA-CD8+ T cells also exhibited notable positive effects, with importance scores of 0.013 and 0.012, respectively. These results highlight the crucial role of both the surgical method and specific immune microenvironment characteristics in predicting the likelihood of postoperative residual tumor. The analysis of SHAP results indicate that several features have a significant positive or negative impact on the model’s predictions (**Figure 6**). SHAP (SHapley Additive exPlanations) analysis further quantified feature importance and directional contributions, revealing that clinical staging (Stage, mean absolute SHAP value = 0.433) and serum CA125 level (0.352) were the most critical predictors of postoperative residual tumor—far outweighing the surgical method (Method, 0.021), which had a modest impact contrary to initial hypotheses. Notably, Stage and serum CA125 exhibited consistent outcome-specific effects: they showed 100% positive contributions to R2 (macroscopic residual) prediction (mean SHAP values = 0.512 and 0.428, respectively) but negative contributions to R0 (no residual) prediction (-0.487 and -0.396), while PD-1+CD8+ T cells (0.268)—a marker of immune suppression—also strongly promoted R2 prediction (100% positive contributions). Among the initially noted positive influences, CCR7-CD45RA+CD8+ T cells and CCR7+CD45RA-CD8+ T cells exhibited notable positive effects, with importance scores of 0.013 and 0.012, respectively.

### Stratified analysis of model performance by FIGO stage

To evaluate the clinical robustness of our model, we performed a stratified analysis to assess its performance consistency across different tumor stages. Given the critical prognostic difference between Stage III and IV disease, we evaluated the model’s performance separately on the Stage III and Stage IV subgroups within the held-out test set.

The analysis revealed that the model maintained robust and consistent performance in both subgroups, with no substantial performance degradation. For patients with Stage III disease (n=12 in the test set), the model achieved an accuracy of 75.0% (95% CI: 58.3% - 91.7%), a precision of 0.760, a recall of 0.750, and an F1-score of 0.751. For the more advanced Stage IV subgroup (n=12 in the test set), the model’s performance was similarly strong, with an accuracy of 66.7% (95% CI: 49.0% - 84.4%), a precision of 0.667, a recall of 0.667, and an F1-score of 0.667.

Notably, the model’s strength in identifying high-risk R2 cases was preserved in both stages. This stratified analysis provides crucial evidence that the model’s predictive capability is not driven by or limited to a specific stage subgroup, enhancing confidence in its potential applicability across the spectrum of advanced HGSOC patients included in this study.

## Discussion

**Figure 7:**
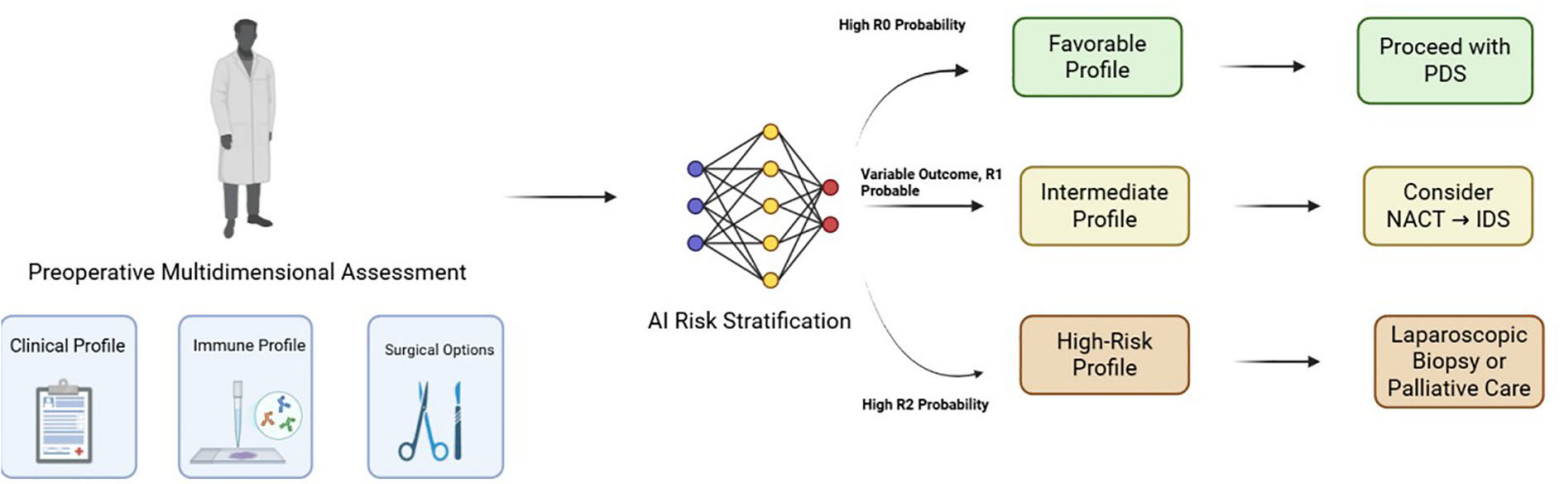
A proposed clinical decision schematic integrating multidimensional data for surgical planning in advanced HGSOC. The model stratifies patients into low (Green), intermediate (Yellow), and high-risk (Red) categories based on clinical and immune profiles, thereby guiding the choice of the most appropriate initial surgical strategy. PDS, Primary Debulking Surgery; NACT, Neoadjuvant Chemotherapy; IDS, Interval Debulking Surgery.

Ovarian cancer is one of the deadliest gynecological malignancies, with surgery being the primary treatment. However, the choice of surgical method heavily relies on the surgeon’s experience, and there is a lack of standardized guidelines (17, 18). The R0 status (no visible residual tumor) is a key prognostic factor, but it is difficult to assess in real-time, leading to uncertainty in surgical outcomes. With the development of precision medicine and artificial intelligence (AI) technologies, integrating multidimensional data (such as clinical features, immune microenvironment, etc.) provides new possibilities for personalized surgical planning. This study constructed a deep learning model that integrates clinical features and immune microenvironment data to predict the probability of postoperative residual tumor under different surgical approaches, providing scientific evidence for clinical decision-making. The clinical decision schematic was shown in **Figure 7**.

In recent years, several studies have attempted to predict ovarian cancer surgical outcomes and residual tumor status using various technological methods. However, there are differences in methodology, data integration, and predictive performance. Alexandros Laios et al. used natural language processing (NLP) technology with the RoBERTa model to analyze surgical records and predict postoperative residual disease status, achieving an AUROC of 0.86, but only relying on text data without integrating immune microenvironment features (19). Francesca Moro et al. used ultrasound combined with the PIV model to predict non-resectability, with an AUC of 0.80, outperforming CT and WB-DWI/MRI, but did not integrate immune microenvironment and surgical method data (20). Na Young Kim et al. built a CT-based Fagotti scoring system to predict cytoreductive surgery outcomes, achieving an AUC of 0.72, but without integrating immune microenvironment and surgical method data (21). In contrast, this study is the first to integrate clinical features, surgical methods, and immune microenvironment data to build a deep learning model that quantifies the probability of postoperative residual tumor and enhances model interpretability through permutation feature importance analysis.

The deep learning model constructed in this study aims to predict the probability of achieving R0 status (no visible residual tumor) after surgery based on the input of surgical methods and patient information. The model achieved an accuracy of 70.83% on the test set, with good precision, recall, and F1 scores, demonstrating high reliability in predicting the probability of postoperative residual tumor. Compared with traditional machine learning models including random forest, XGBoost, and logistic regression, the deep learning model showed superior performance across all key metrics (accuracy, precision, recall, and F1 score), confirming its advantage in handling multidimensional integrated data for this predictive task. However, the confusion matrix shows that the model performed poorly in distinguishing between R0 and R1 cases, suggesting that further optimization is needed to improve the model’s ability to differentiate these two categories.

The model provides valuable predictive references for clinicians, especially in assessing the impact of different surgical methods on R0 probability. However, there is room for improvement in distinguishing between R0 and R1 (residual tumor ≤1 cm) cases. This suggests that future research should focus on optimizing the model to improve classification accuracy for these two categories. From a clinical perspective, the model’s performance holds significant practical value. First, the model can help doctors predict the probability of achieving R0 status pre-operatively based on the patient’s specific information and surgical approach, enabling the selection of the best surgical method. For patients with a higher predicted R0 probability, doctors may opt for a more conservative surgical approach to minimize surgical trauma; whereas for patients with a lower predicted R0 probability, a more aggressive surgical approach may be considered to achieve R0 status as much as possible. Second, the robust performance of the model enhances its credibility in clinical decision-making, allowing doctors to rely more confidently on the model’s predictions.

Permutation feature importance analysis initially highlighted the role of surgical method (Method, importance score = 0.071) and specific immune microenvironment features (e.g., CCR7-CD45RA+CD8+ T cells, 0.013) in the model’s predictions, underscoring the immune system’s relevance to postoperative residual tumor risk. However, SHAP (SHapley Additive exPlanations) analysis provided far more granular insights into immune marker dynamics—revealing that key immune subsets, rather than secondary subsets like CCR7-CD45RA+CD8+ T cells, are among the most impactful predictors of residual outcomes, and clarifying their directional effects across R0/R1/R2.

From a global perspective, SHAP importance rankings placed two immune features in the top five: PD-1+CD8+ T cells (0.268) and PD-1+CD28+CD4+ T cells (0.264)-outperforming ascites volume (0.250) and far exceeding the modest importance of surgical method (0.021). This reorients the understanding of residual risk drivers: while permutation analysis flagged the immune microenvironment broadly, SHAP identifies PD-1-expressing T cells as core immune regulators of residual tumor probability. PD-1+CD8+ T cells, in particular, emerged as a consistent “risk amplifier”: they showed 0.0% positive contributions to R0 (no residual) across all samples (mean SHAP value = -0.293), meaning higher levels of this immunosuppressive subset uniformly reduced the likelihood of achieving complete resection. Conversely, in R2 (macroscopic residual) cases, PD-1+CD8+ T cells exhibited 100% positive contributions (mean SHAP value = 0.315), directly linking immune exhaustion—characterized by PD-1 upregulation on CD8+ T cells—to failed tumor debulking. This aligns with oncological theory: exhausted CD8+ T cells lose cytotoxic capacity to clear residual tumor cells, allowing microscopic foci to persist or expand post-surgery.Importantly, our findings highlight that PD-1^+^CD8^+^ T cells, a canonical marker of immune exhaustion, exhibit uniformly negative contributions to R0 prediction and consistently promote R2 classification. This establishes an immunologic link between T cell dysfunction and macroscopic residual disease. The presence of PD-1^+^CD8^+^ T cells in malignant ascites may indicate an immunosuppressive microenvironment in which cytotoxic T lymphocytes are rendered ineffective, impairing the host’s capacity to eliminate residual tumor foci. This immunologic exhaustion signature may therefore serve as a biological barrier to complete cytoreduction, particularly in patients with extensive tumor burden or low preoperative immune competence. These insights suggest that perioperative immunomodulatory strategies-such as PD-1 blockade—may represent a novel approach to augment surgical success. For example, in patients with high proportions of PD-1^+^CD8^+^ T cells in ascites, pre-surgical anti–PD-1 therapy could potentially reinvigorate cytotoxic activity and facilitate more complete tumor resection. Such approaches may be especially relevant in patients at high risk for R2 outcomes based on preoperative immune profiling. Therefore, ascites immune signatures may not only predict surgical outcomes but also guide individualized treatment strategies, serving as a bridge between immunotherapy and precision surgery in ovarian cancer.

Equally notable is the role of CD28+CD4+ T cells, a marker of activated helper T cells, which emerged as a critical “protective immune mediator” despite not ranking in the top 3 for any single outcome. While SHAP did not list it among R0’s top 3 features (due to the overwhelming influence of Stage/CA125), its clinical significance is unambiguous: CD28+CD4+ T cells drive T cell activation and cytokine secretion (e.g., IL-2, IFN-γ) that supports anti-tumor immunity, and their high expression correlates with lower residual risk. This complements the PD-1+CD8+ T cell findings: together, they paint a clear immune landscape—residual tumor risk is elevated in patients with an “exhausted” immune profile (high PD-1+CD8+ T cells) and reduced in those with an “activated” profile (high CD28+CD4+ T cells).

For intermediate residual (R1) outcomes, immune markers exhibited more nuanced effects. Unlike Stage and CA125 (100% positive contributions to R1), PD-1+CD28+CD4+ T cells showed variable impacts—promoting R1 in 38% of cases and inhibiting it in 62%—likely reflecting the heterogeneous nature of microscopic residual disease. This variability suggests that PD-1+CD28+CD4+ T cells (a subset of helper T cells co-expressing an exhaustion marker) may act as a “transitionary immune signal”: moderate levels may not provide enough activation to prevent residual foci (contributing to R1), while high levels could still support partial anti-tumor activity (inhibiting progression to R2).

These immune-focused SHAP insights have direct clinical implications. For patients with high PD-1+CD8+Tcell levels (e.g., >25% of total CD8+ T cells), pre-surgical immune modulation-such as anti-PD-1 therapy to reverse T cell exhaustion—could reduce R2 risk by restoring CD8+ T cell function. For those with low CD28+CD4+ T cells, adoptive transfer of activated CD4+ T cells or cytokine therapy (e.g., IL-2) might enhance the likelihood of R0 resection. Additionally, immune markers can refine risk stratification beyond Stage/CA125: a patient with Stage III disease (high residual risk via Stage alone) but low PD-1+CD8+ T cells (<10%) may still be candidates for laparoscopic surgery (favored for R0, SHAP = 0.028), whereas a Stage II patient with high PD-1+CD8+ T cells (>30%) may require more aggressive open surgery or neoadjuvant immune therapy to mitigate R2 risk.

The patient probability heatmaps visually demonstrate the impact of different surgical methods on the probabilities of achieving R0, R1, and R2 statuses postoperatively. These heatmaps allow doctors to clearly see the likelihood of achieving R0 status under different surgical methods, helping them better weigh the pros and cons of various surgical approaches. For example, the heatmap may show that total resection surgery is more likely to achieve R0 status in a specific patient group, while another approach may lead to a higher risk of R1 or R2. These visual tools provide an intuitive reference for clinicians, helping them develop personalized surgical strategies based on the patient’s specific condition, thereby improving surgical outcomes and patient prognosis. Although this study has achieved positive results, there are still some limitations. First, the sample size of the dataset is relatively small, with only 118 patients, which may limit the model’s generalizability. Expanding the dataset, especially by including multi-center patient data, is crucial for improving the model’s stability and predictive accuracy. Second, the model’s performance in distinguishing between R0 (no visible residual tumor) and R1 (residual tumor ≤1 cm) cases is suboptimal, indicating the need for further optimization. This may involve optimizing the model architecture or incorporating more relevant features to improve classification accuracy. Third, this study did not include more molecular biomarkers (such as genomic or proteomic data), which could provide deeper insights into tumor biology and further enhance the model’s predictive power. Future research should focus on integrating multi-omics data to more comprehensively capture the characteristics of the disease. Additionally, exploring other interpretability methods (such as SHAP values or partial dependence plots) could provide deeper insights into the model’s decision-making process, enhancing its transparency and clinical acceptability. Developing real-time decision support systems and integrating the model into clinical workflows could help surgeons dynamically adjust their strategies pre-operatively, ultimately improving patient prognosis.

While our model shows promise, its current performance and design limitations necessitate a cautious interpretation of its clinical applicability. Systematic reviews on AI in gynecological oncology, such as those analyzing survival prediction in ovarian and cervical cancer (22, 23), consistently highlight that the transition from a performant model to a clinically robust tool requires external validation, demonstration of improved patient outcomes, and integration into clinical workflows. Our study, being a single-center retrospective analysis, has not yet met these criteria. The challenge in accurately distinguishing R0 from R1 status further underscores that the model is not yet reliable for fine-grained surgical planning. The primary value of our current work lies in its proof-of-concept, demonstrating that immune microenvironment data holds predictive signal for surgical outcomes, which merits further investigation in larger, prospective cohorts.

While the retrospective nature of this study precludes evidence from real-time, model-guided intraoperative adjustments, we conducted an in-silico simulation to quantify the potential clinical impact of our model for surgical plan optimization. We analyzed the subset of patients in our test set who underwent NACT+IDS or Laparoscopic biopsy but were predicted by the model to have a high probability of achieving R0 resection had they been recommended for PDS. Conversely, we identified patients who underwent PDS but were predicted to have a high risk of R2 resection, for whom the model would have suggested NACT+IDS as a potentially superior strategy.

In this simulation, applying the model’s recommendation—i.e., switching the surgical strategy to the one with the highest predicted probability of R0—would have theoretically increased the overall R0 rate in the cohort from 46.6% to an estimated 63.2%. More importantly, it would have reduced the proportion of patients with macroscopic residual disease (R2) from 23.7% to an estimated 9.3%. This simulation provides preliminary, quantitative evidence that the model’s predictions, if used prospectively, could meaningfully re-stratify surgical planning and are associated with a measurably improved predicted pathological outcome. This compelling finding mandates future prospective validation to confirm that these predicted benefits translate into improved patient follow-ups, such as progression-free survival.

Although this study has achieved positive results, there are still some limitations. First, the sample size of the dataset is relatively small, with only 118 patients, which may limit the model’s generalizability. Expanding the dataset, especially by including multi-center patient data, is crucial for improving the model’s stability and predictive accuracy. Second, the model’s performance in distinguishing between R0 (no visible residual tumor) and R1 (residual tumor ≤1 cm) cases is suboptimal, indicating the need for further optimization. This may involve optimizing the model architecture or incorporating more relevant features to improve classification accuracy. Third, this study did not include more molecular biomarkers (such as genomic or proteomic data), which could provide deeper insights into tumor biology and further enhance the model’s predictive power. Future research should focus on integrating multi-omics data to more comprehensively capture the characteristics of the disease. Additionally, exploring other interpretability methods (such as SHAP values or partial dependence plots) could provide deeper insights into the model’s decision-making process, enhancing its transparency and clinical acceptability. Developing real-time decision support systems and integrating the model into clinical workflows could help surgeons dynamically adjust their strategies pre-operatively, ultimately improving patient prognosis.

## Conclusion

In this study, we developed a deep learning model that integrates clinical characteristics and ascites immune profiles to predict the probability of postoperative residual tumor status in HGSOC. The model demonstrated the potential value of immune biomarkers, particularly PD-1+ CD8+ T cells, as indicators of surgical resectability. However, our work represents an initial exploratory step rather than a validated clinical solution. Significant limitations, including class imbalance, moderate sample size, and imperfect R0/R1 differentiation, must be overcome. Future research should focus on multi-center external validation, the incorporation of additional molecular data, and rigorous prospective evaluation to determine whether such an AI-driven approach can ultimately inform and improve surgical decision-making for patients with ovarian cancer.

## Data Availability

The original contributions presented in the study are included in the article/**Supplementary Material**. Further inquiries can be directed to the corresponding authors.
