# Supplementary figures and images for: A multidimensional data-driven approach to surgical plan optimization and postoperative residual tumor prediction in ovarian cancer
Source: Front Immunol. 2025 Dec 17;16:1705428. doi: 10.3389/fimmu.2025.1705428 (PMC12753436; doi:10.3389/fimmu.2025.1705428)

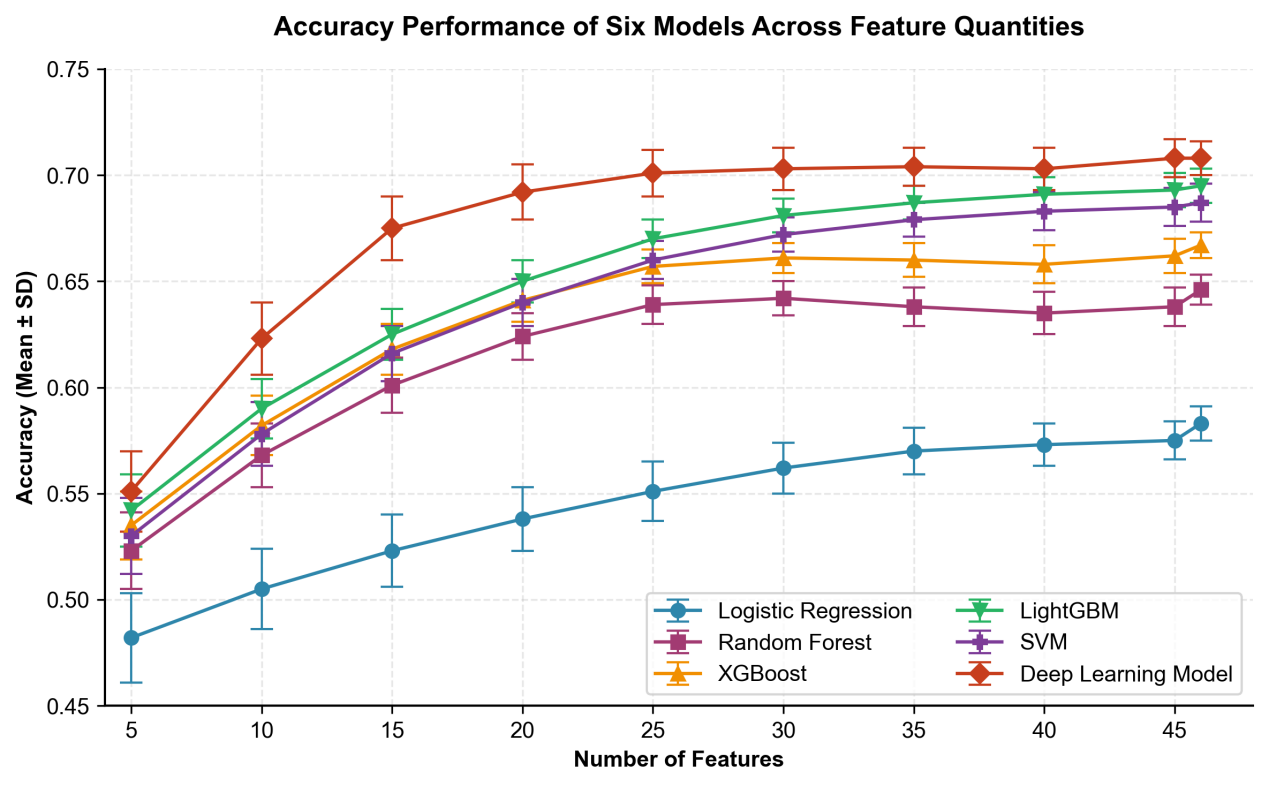


Figure S1. The accuracy performance comparison across feature quantities.

Supplement: Supplementary file 1 [file DataSheet1.docx]
